# Aluminium Solder

**Published:** 1868-11

**Authors:** 


*Aluminium Solder*—The great difficulty in working aluminium has at length been overcome by the discovery of a solder composed of aluminium seven-eighths and tin one-eighth, the formula of Dr. A. Starr.

By the use of such a solder, swaged aluminium plates can be made and the teeth attached by a process similar to that of mounting teeth upon gold plate, saving much of the time and labor required by the cast aluminium base. Dr. Starr's solder has been tested by a large number of dentists, the majority of whom speak of it in the highest terms; care and skill, however, are required in its manipulation. Drs. B. W. Franklin and A. P. Preterre have also produced solders for aluminium, which they claim will withstand the action of the fluids of the mouth.

